# Sustainable Development of Urban Metro System: Perspective of Coordination between Supply and Demand

**DOI:** 10.3390/ijerph181910349

**Published:** 2021-09-30

**Authors:** Yinghan Zhu, Liudan Jiao, Yu Zhang, Ya Wu, Xiaosen Huo

**Affiliations:** 1 School of Economics and Management, Chongqing Jiaotong University, Chongqing 400074, China; zhuyinghan@mails.cqjtu.edu.cn (Y.Z.); yuzhang@cqjtu.edu.cn (Y.Z.); huoxiaosen@cqjtu.edu.cn (X.H.); 2 College of Resources and Environment, Southwest University, Chongqing 400715, China; mswuya@swu.edu.cn

**Keywords:** metro system, indicator system, coupling coordination degree, TOPSIS, demand and supply

## Abstract

Metro systems are gradually becoming more and more crucial in promoting the economy and society in cities. However, various challenges such as financial resources and the efficiency of utilizing these metro plans bring difficulties for metro construction. Hence, accurately evaluating the urban metro system’s development condition seems significant for the sustainable development of the urban metro system. Therefore, a comprehensive evaluation indicator system of metro development conditions containing 25 indicators from dimensions of demand and supply is established in this study, and a coupling coordination degree model combined with the entropy weight method and Technique for Order Preference by Similarity to an Ideal Solution (TOPSIS) method is proposed to analyze the level of metro development conditions and coupling coordination conditions of 35 cities in China. According to the calculation results, 35 cities are divided into six categories, and radar charts are constructed to promote the sustainable development of the metro system.

## 1. Introduction

As the most critical component in urban infrastructures, the metro system plays a crucial role in promoting a city’s economy and society development. Compared with other modes of transportation, such as buses, taxis, and private cars, the metro system has many advantages and benefits, such as environment protection, better travel safety and efficiency, and high travel punctuality [1,2], while raising a positive stimulating effect on urban transport planning, intelligent transport system, and energy resources [1,3,4,5], so, more and more countries are willing to develop metro construction. By the end of 2020, 178 cities from 57 nations, mainly in Asian areas, European areas, and North American areas, had built metro systems, with a total length of 17,584.77 km and 12,567 stations [6]. As the biggest developing country globally, China has also been building metro systems, particularly in its big cities, to solve traffic congestion induced by the growth of the urban population. It was reported by the China Association of Metros (2021) that, by the end of 2020, 45 cities had built metro systems in China, with a total length of 7969.7 km and a total number of 244 lines in the whole country [7]. Twenty additional cities are planning for building metro systems. In total, about 7085.5 km of new metro lines will be built in China in the future.

However, it is possible that these ambitious metro plans will encounter many challenges, such as financial resources and the efficiency of utilizing these metro plans. It is appreciated that considerable investment is needed to build the metro system. For example, it is reported that the average cost of metro construction rose to 800 million CNY per kilometer in urban areas in China [8]. Furthermore, operating the metro system costs are higher than the construction costs from the whole system lifecycle. Some city governments cannot even afford the operation costs, although they can construct the metro system. In this case, the city government has to provide support to cover high operation costs. According to the statistic data from 2007 to 2014 from the Beijing Municipal Finance Bureau, the operation of the Beijing metro system is primarily supported by the fiscal subsidy, accounting for more than 5% of the revenue of the Beijing government. Therefore, the finance resource is a big challenge to implement the 7085.5 km metro systems planned in China’s future years. On the other hand, the efficiency of using metro systems is different between different cities as they have a different scale of the metro system and management skills. Some cities have the problem of shortage of the metro system, and others face oversupply. For example, it was found that the scale of the metro system in Kunming has over-supply capacity [9], indicating that the metro system in this city has low utility efficiency. Meanwhile in other cities, such as Beijing, the scale of the metro system is undersupplied, evidenced by the crowded ridership.

According to the definition of sustainable development, it emphasizes the ability to meet society’s current needs without compromising the capacity to satisfy future generations’ demands [10]. In addition, various dimensions of objectives, including economy, society, environment, resources, must be coordinated and developed in harmony [11]. The metro system’s sustainable development also needs to solve various problems such as financial resources, utility efficiency, and other natural hazards associated with urban mass transport systems [12]. Therefore, it is crucial to correctly evaluate the urban metro system’s development condition for the sustainable development of the urban metro system. For the development process for the metro system, as pointed out in other studies, the factors affecting the development condition of the metro system can be summarized from the perspective of supply and demand [13]. Specifically, the supply of the metro system is the support of human, material, and financial resources that a city can provide for the development of the metro system. For instance, Loo et al. [3] proposed the benchmarks to evaluate the readiness of a city for metro development from the perspective of economy, which is that a city with a Gross Domestic Product (GDP) of 74,080 CNY per capita can be identified as the city ready for building metro lines. In addition, Sharav et al. [4] emphasized the importance of assessing the investment accurately for metro construction by demonstrating a methodological approach. Besides economy, technology is also considered necessary support for metro construction consisting of hardware and software [14]. The demand for the metro system refers to the purpose that a city needs to build a metro system to reduce urban traffic congestion, environmental pollution, and citizens’ travel demand. For example, The National Development and Reform Commission in China (2018) advised that the local governments must consider some critical parameters when planning the metro system network, including the distribution of population within a city, the city traffic demand, land use and others. Loo et al. [14] pointed out that population, space constraint, cost constraint, and environmental considerations all encouraged the development of public transport networks such as metro systems. Moreover, fares, quality of service, and car ownership also contribute to the development of the metro system from the perspective of demand [15,16]. Among them, fares and quality of service belong to the attributes of the metro system, which can influence demand directly because higher fares or worse quality of service will cause lower passenger flow volume. At the same time, it is also considered that if an increase in car ownership occurs, the demand for the metro system will be reduced [16].

Therefore, there is a need for coordinated development between supply and demand conditions for developing the metro system. It is considered very important to plan the scale of metro systems properly. Suppose the scale of the metro system is too small compared to the population scale of a city. In that case, it will not meet the transportation demand of the urban population [17]. On the other hand, if a metro system is too big, that is oversupply, there will be low utility efficiency, which will result in the consequences of investment waste and the waste of land resources [17,18]. Different transport system models are raised to explore the relationship between demand and supply in urban mass transport. For example, Chen et al. [19] identified the relative spatial gaps in public transport supply and demand from seniors by calculating the public transport supply and public transport demand indices. Hörcher and Graham [20] investigated public transport supply conditions when facing independent but nonidentical demand conditions using a baseline model based on the crowding multiplier approach and the crowding cost function. Konečný et al. [21] concluded that the supply of connections is not a statistically significant indicator that refers to demand in some cases using correlation analysis and covariance methods. In addition, Malavenda et al. [22] simulated the two-way relationship between land use and transport systems in urban areas based on Land Use and Transport Interaction (LUTI) models. Croce et al. [23] constructed the path and route choice model of commercial vehicles travelling on a road network due to the demand conditions.

Although previous studies have paid attention to the supply and demand of the metro system by using various transport system models from different perspectives, few studies focus on the coordination problem of the demand and supply. In fact, the coupling coordination degree model has vast advantages in studying the coordination problem. Coupling, a concept in physics, refers to the phenomenon that several physical systems interact with each other through internal connection, so coupling degree is proposed to describe the degree of interaction among systems [24]. In addition, the coordination degree is raised to measure the status or level of coordination of systems. Therefore, combining the concepts of coupling degree and coordination degree, the coupling coordination degree is used to measure whether the internal systems are in harmony with each other in the development process, considering interaction and coordination [25]. The coupling coordination degree has been widely used to analyze urban development, and most studies concentrate on the subsystems of the whole city, especially between economy and ecology. For example, Sun and Cui [26] applied a coupling coordination degree model to analyze the development in harmony with the economic, social, and environmental benefits of urban public transportation infrastructure to improve the coordinated development level. Li and Yi [27] evaluated the sustainability of cities by calculating coordination among economy, society, and environment with the coupling coordination degree model. Shen et al. [28] introduced an improved coupling coordination degree model to evaluate the coordination between socio-economy and carbon emissions for sustainable urban planning. Xing et al. [29] assessed the coordinated results in the economy-resource-environment system based on the coupling coordination degree model for sustainable urban development.

The above discussions demonstrate a small existing study assessing the coupling coordination degree of metro systems from a perspective between supply and demand conditions. Therefore, in this study, an assessment model is developed to evaluate the coupling coordination degree between the supply and demand conditions for developing the metro system. And then, the coupling coordination conditions of 35 cities in China are analyzed. The contributions of this paper are as follows. First, while some scholars have researched demand or supply conditions of the metro system, previous studies mainly concern the factors affecting development conditions from the perspective of demand or supply [3,4,14,15,16], and empirical analysis of demand or supply conditions in the background of seniors [19], independent but nonidentical demand conditions [20], and normal cases [21]. In this study, under the background of sustainable development, coupling coordination conditions between demand and supply can provide a new angle for developing the metro system. Second, in the case study results, the categories of the coupling coordination degree of the metro system between demand and supply are identified. The suggestions to improve the development of the metro system are also discussed. The research results in this study can provide a reference for other coupling coordination problems in sustainable urban development.

## 2. Construction of the Metro Development Conditions Comprehensive Evaluation Indicator System

Demand can be defined as the amount of all services and products currently utilized, consumed, or expected to be obtained by the human society in a particular area [30]. Similarly, the demand for the metro system means the number of metros to adapt to the conditions currently, even in the future. After collecting and consolidating the literatures of factors affecting metro system demand [3,4,14,15,16,31], four dimensions, including satisfaction of people’s going out (D1), improvement of urban environmental conditions (D2), improvement of urban traffic conditions (D3), and perfection of the development of metro (D4), are proposed to reflect the level of the demand for the metro system. Among them, the satisfaction of people’s going out mainly considers the amount and the density of population because a higher amount or density will lead to the possibility that more metro systems are needed to accommodate the passengers. As for the improvement of urban environmental conditions, compared to other modes of transportation, the metro system can reduce both exhaust pollution and noise pollution from cars, significantly contributing to the people’s living environment. In the dimension of improving urban traffic conditions, taking road facilities and other transportation infrastructures into account, the metro system can reduce the traffic congestion and the pressure of operation of mass traffic. Finally, the metro’s current construction condition and operational condition can directly report whether the metro system needs to be continuously constructed. Therefore, through a further analysis on the relevance, comparability, and data availability of indicators, 16 demand indicators are selected, as shown in Table 1.

Supply refers to the capacity to provide services and goods for human society [30]. Similarly, the supply of the metro system can be defined as the services and goods that the local government or city can provide. Through considering the relevant references [3,4,14,15,16,31], three dimensions, including finance (S1), manpower (S2), and land (S3), are proposed to reflect the level of supply for the metro system. Finance (S1) and manpower (S2) represent the expense and workers that the government can afford or offer in the construction and operation of the metro system. A more excellent level of finance or manpower means a more outstanding development environment in terms of supply conditions. Land (S3) can provide the space for construction and the passenger flow for the operation indirectly. Hence, through a further analysis on the relevance, comparability, and data availability of indicators, 9 supply indicators are selected, as shown in Table 1.

## 3. Methodology and Research Materials

**Figure 1:**
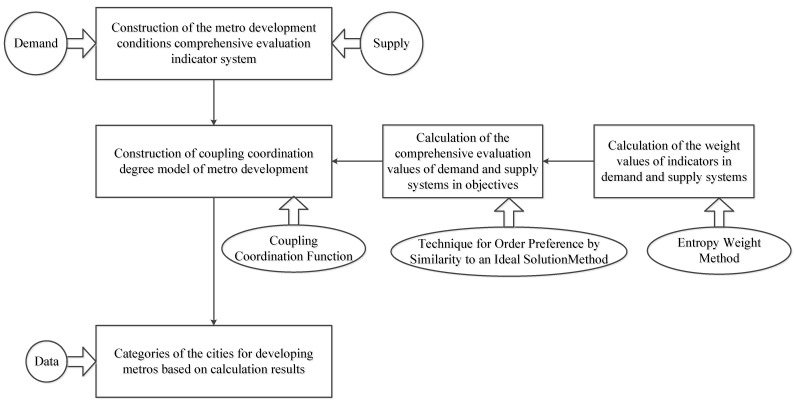
Producers of methodology.

In order to measure the coupling coordination degree of metro systems, three procedures are proposed in Figure 1. Firstly, a comprehensive evaluation indicator system of metro development conditions is carried out from demand and supply perspective. Secondly, a coupling coordination degree model is developed based on the coupling coordination function to assess the coupling coordination conditions of metro systems. Furthermore, two other methods are also used in the model. The Technique for Order Preference by Similarity to an Ideal Solution (TOPSIS) method is used to calculate the comprehensive evaluation values of demand and supply systems in metro systems. The entropy weight method is used to calculate the weight values of indicators in demand and supply systems. Finally, through data collection, an empirical analysis can be raised, and the categories of the cities for developing metros based on calculation results can be identified.

### 3.1. Development of Coupling Coordination Degree Model

#### 3.1.1. Coupling Coordination Function

This study uses coupling coordination degree (*D*) to evaluate the interaction between demand and supply systems and reflect the dynamic trend of coordination and transverse comparison between demand and supply systems [40]. Coupling coordination degree is a quantitative index ranging from 0 to 1. The higher value indicates the more coordinated metro development. The index has been used in oasis urbanization [40] and urban infrastructure [41].

According to Zhang et al. [42], coupling degree (*C*) and coordination degree (*T*) is used to calculate the coupling coordination degree (*D*), and the basic equation of coupling coordination degree can be presented as follows:

(1) $$D\;=\;\sqrt{C\;\times \;T}$$

where *C* represents the coupling degree of the metro system, and *T* represents the coordination degree of the metro system.

In Equation (1), coupling degree (*C*) comes from physics. It can be defined as a phenomenon in which the systems under investigation influence each other through different links [10]. The basic equation to calculate coupling degree (*C*) can be presented as follows [10,24,25]:

(2) C = x{(u1×u2×⋯×ux)[∏(u1+u2+⋯+ux)]}1x

where *x* denotes the number of systems and *u_x_* represents the comprehensive development value of *x-th* system.

In this study, there are two systems of the metro system: the demand system and the supply system. Therefore, in Equation (2), the value of *x* is 2, and Equation (2) can be simplified as:

(3) $$C\;=\;2\sqrt{\frac{u_{d}\times u_{s}}{{\left(u_{d}+u_{s}\right)}^{2}}}$$

where *u_d_* represents the comprehensive development value of the demand system, and *u_s_* represents the comprehensive development value of the supply system.

In Equation (1), coordination degree (*T*) refers to the degree of benign coupling in the coupling interaction relationship, which reflects the quality of coordination among systems [42]. The basic equation to calculate coordination degree (*T*) can be presented as [42]:

(4) $$T\;=\;\alpha u_{1}+\beta u_{2}+\cdots +\gamma u_{x}$$

where *x* denotes the number of systems, and *u_x_* represents the comprehensive development value of *x*-th system. In addition, *α*, *β* and *γ* represent the degree values of the importance of systems. 

In this study, there are two systems of the metro system: the demand system and the supply system. Therefore, in Equation (4), the value of *x* is 2. Meanwhile, the same importance of the demand and supply system is considered. Therefore, in Equation (4), the value of *α* and *β* is 0.5. The Equation (4) can be simplified as:

(5) $$T\;=\;\frac{1}{2}u_{d}+\frac{1}{2}u_{s}$$

where *u_d_* represents the comprehensive development value of the demand system, and *u_s_* represents the comprehensive development value of the supply system.

By substituting the results Equation (3) and (5) into Equation (1), the coupling coordination degree is presented as follows:

(6) $$D\;=\;\sqrt{C\;\times \;T}\;=\;\sqrt{\frac{2\sqrt{u_{d}\times u_{s}}}{u_{d}+u_{s}}\times \frac{1}{2}(u_{d}+u_{s})}\;=\;\sqrt{\sqrt{u_{d}\times u_{s}}}\;=\;{\left(u_{d}+u_{s}\right)}^{\frac{1}{4}}$$

According to the relative research, the coupling coordination degree is divided into 4 levels and 12 types in this study, as shown in Table 2 [29,35].

#### 3.1.2. TOPSIS Method

The comprehensive evaluation includes three main steps: selecting the indicator system, calculating indicators’ weights, and selecting evaluation methods. The selection of evaluation methods is the core stage in the whole process, which is also a significant way to obtain the conclusion. Generally, these evaluation methods are classified into parametric and non-parametric. Moreover, Data Envelopment Analysis (DEA) and Multi Criteria Decision Making Methods (MCDM) are the most commonly used methods in recent years. DEA is a non-parametric method that concentrates on evaluating decision-making units’ efficiency [43,44], and MCDM emphasizes the comprehensive rankness of objectives [45]. As one method of the MCDM, TOPSIS makes full use of objective data and outputs the evaluation conditions with accurate numerical values, which adapts to the research aim of this study.

The TOPSIS method, proposed initially by Hwang and Yoon [46], is used to get the comprehensive development values of the demand (*u_d_*) and supply system (*u_s_*) in this study, which constructs the positive and negative ideal solutions of multi-index problems, considering the proximity to the positive ideal solution, and the distance from the negative ideal solution as the basis for evaluating feasible solutions [47]. Moreover, the method has been used in urban public transport [48], city economy [49], and smart city [50]. The specific steps are as follows [32,47]:

Step 1. Establishment of an evaluation matrix

With *n* indicators to measure the development of metro systems in *m* cities, the evaluation matrix can be expressed as

(7) $$Y\;=\;\left[\begin{array}{cccc} y_{11} & y_{12} & \cdots & y_{1n}\\ y_{21} & y_{22} & \cdots & y_{2n}\\ \vdots & \vdots & \ddots & \vdots \\ y_{m1} & y_{m2} & \cdots & y_{mn} \end{array}\right]$$

where *y_ij_* represents the evaluation value of the *j*-th evaluation indicator in the *i*-th city.

Step 2. Normalization of all indicators

The evaluation indicators are divided into positive indicators and negative indicators. For those positive indicators, that is, a larger value indicates a better result. The normalized value can be calculated as follows:

(8) $${y^{\prime }}_{ij}\;=\;\frac{y_{ij}-\min(y_{j})}{\max(y_{j})-\min(y_{j})}$$

For those negative indicators, a smaller value indicates a better result. The normalized value can be calculated as follows:

(9) $${y^{\prime }}_{ij}\;=\;\frac{\max(y_{j})-y_{ij}}{\max(y_{j})-\min(y_{j})}$$

where $\min(y_{j})$ and $\max(y_{j})$ are the minimum value and maximum value for the indicator *j*. And *y*′*_ij_* represents the evaluation value of the *j*-th evaluation indicator in the *i*-th city after normalization.

Step 3. Construction of a weighted decision matrix

The weighted decision matrix can be obtained through the product of the matrix after normalization and the entropy weight as follows:

(10) $$Z\;=\;\left[\begin{array}{cccc} z_{11} & z_{12} & \cdots & z_{1n}\\ z_{21} & z_{22} & \cdots & z_{2n}\\ \vdots & \vdots & \ddots & \vdots \\ z_{m1} & z_{m2} & \cdots & z_{mn} \end{array}\right]$$

(11) $$z_{ij}\;=\;{y^{\prime }}_{ij}\times \omega_{j}$$

where *z_ij_* represents the weighted evaluation value of the *j*-th indicator in the *i*-th city after normalization. And $\omega_{j}$ represents the weight of the *j*-th evaluation indicator.

Step 4. Calculation of the positive ideal distance and the negative ideal distance

Before calculating the positive ideal distance and the negative ideal distance, the positive ideal value $\left({Z^{+}}_{ij}\right)$ and the negative ideal value $\left({Z^{-}}_{ij}\right)$ of the *j*-th evaluation indicator in the *i*-th city should be calculated as follows:

(12) $${Z^{+}}_{ij}\;=\;\left\{\max Z_{ij}|i=1,2,\cdots ,m\right\}\;=\;\left\{{Z^{+}}_{i1},{Z^{+}}_{i2},\cdots {Z^{+}}_{in}\right\}$$

(13) $${Z^{-}}_{ij}\;=\;\left\{\min Z_{ij}|i=1,2,\cdots ,m\right\}\;=\;\left\{{Z^{-}}_{i2},{Z^{-}}_{i2}\cdots {Z^{-}}_{in}\right\}$$

Then, based on the positive ideal value and the negative ideal value, the positive ideal distance $\left({d^{+}}_{i}\right)$ and the negative ideal distance $\left({d^{-}}_{i}\right)$ of the *i*-th city should be calculated as follows:

(14) $${d^{+}}_{i}\;=\;\sqrt{{\sum_{j=1}^{n}\left(Z_{ij}-{Z^{+}}_{ij}\right)}^{2}},i=1,2,\cdots m$$

(15) $${d^{-}}_{i}\;=\;\sqrt{{\sum_{j=1}^{n}\left(Z_{ij}-{Z^{-}}_{ij}\right)}^{2}},i=1,2,\cdots m$$

Step 5. Calculation of relative closeness

The relative closeness value (*R_i_*) of the *i*-th city is used to sort the evaluation objects. A larger relative closeness value represents a smaller distance between the evaluation object and the ideal sample. The specific equation is as follows:

(16) Ri = d−id+i+d−i

Therefore, in this study, for the *i-th* city, the comprehensive development values of the demand system (*u_d_*) and supply system (*u_s_*) are represented by the relative closeness values (*R_i_*), respectively.

#### 3.1.3. Entropy Weight Method

The entropy weight method is used to determine the weight of each indicator (*ω_j_*) in this study. Entropy is a measure of the degree of disorder of the system. Suppose the entropy of the indicator is small. In that case, more information is provided by the indicator, which means the role of this indicator in the comprehensive evaluation is bigger. Therefore, the indicator should be given a higher weight [51]. The entropy weight method can effectively avoid the influence of human’s subjective impressions on the evaluation results. At present, the method has been widely applied to many fields such as urban rail transit system operation [36], sustainable performance of urbanization [52], eco-industrial parks [53]. The specific steps are as follows [32,52]:

As for the evaluation matrix after normalization, firstly, the contribution value (*p_ij_*) of *j*-th indicator in *i*-th city should be calculated as follows:

(17) $$p_{ij}\;=\;\frac{{y\prime }_{ij}}{\sum_{i=1}^{m}{y\prime }_{ij}}$$

Then, before obtaining the entropy weight of *j-th* indicator, an entropy value (*e_j_*) for each indicator can be calculated as follows:

(18) $$e_{j}\;=\;-\frac{1}{\ln m}\sum_{i=1}^{m}p_{ij}\ln p_{ij}$$

Finally, the entropy weight value (*ω_j_*) of *j*-th indicator can be calculated as follows:

(19) $$\omega_{j}\;=\;\frac{1-e_{j}}{n-\sum_{j=1}^{n}e_{j}}$$

### 3.2. Data Sources

In this study, 35 cities that developed and operated metro systems in 2018 in China are selected for the empirical analysis. The research data in assessing urban environmental conditions are collected from Ecology and Environment Statement in 2019 of each city in China. And the ones in the perfection of the metro development come from the Annual Statistics and Analysis Report in 2019 of Urban Rail Transit [54]. Moreover, others are from Statistical Yearbook in 2020 of each city respectively [55]. The 35 cities’ values of 25 indicators are shown in Appendix A.

## 4. Analysis Results

### 4.1. Results of Entropy Weights

Firstly, the normalized values of 18 positive and 7 negative indicators in 35 cities are obtained using Equations (8) and (9). After that, the contribution value, the entropy value and the entropy weight of each indicator of demand and supply systems are calculated by the Equations (17)–(19). The entropy weights of the two systems are presented in Table 3 and Table 4.

From Table 3, 16 indicators are in the demand system, and the average weight is 0.0625. In addition, the weight of average daily passenger travel volume stands in the first place, and taxis per 10,000-person locates at the end, whose values are 0.2134 and 0.0192, respectively. Among them, there are 5 indicators whose weight is larger than the average weight. They belong to two-factor layers, which influence the metro development more from the demand dimension. Furthermore, among the four factor layers, the perfection of the development of metro affects the metro development most, followed by the satisfaction of people’s going out and improvement of urban environmental conditions, and improvement of urban traffic conditions takes the last place.

From Table 4, the average weight of 9 indicators is 0.1111. The maximum weight belongs to general public budget revenue and the minimum belongs to the population urbanization rate, whose values are 0.2009 and 0.0258. Moreover, five indicators whose weight is larger than the average weight are distributed in all the factor layers. Therefore, three-factor layers all play an irreplaceable role in developing the metro from the supply dimension.

### 4.2. Results of TOPSIS Values

Next, the weighted values of the two systems are calculated by the Equation (11), and each indicator’s maximum and minimum values in the 35 cities are identified. Therefore, the positive ideal distance, the negative ideal distance, and the relative closeness value of each city of two systems are calculated by the Equations (12)–(16). The results are presented in Table 5 and Table 6.

From Table 5, in the demand system, the average relative closeness value of 35 cities is 0.2950. Shanghai stands in first place with 0.7401, followed by Beijing and Guangzhou, with distinct advantages. In the other cities, the relative closeness values of 14 cities are above the average. The remaining cities are between 0.1607 and 0.2694.

From Table 6, the average relative closeness value of 35 cities is 0.2781 in the supply system. Furthermore, Shanghai, Beijing, Chongqing, Guangzhou, Shenzhen, and Tianjin are located in the top six. And the relative closeness values of the rest cities are between 0.0619 and 0.4554.

### 4.3. Results of Coupling Coordination Degree Values

Finally, by combining the value of *u_d_* in Table 5 and *u_s_* in Table 6 to Equation (6), the coupling coordination degree values of 35 cities are calculated. Taking the Shanghai metro system as an example, the value of *u_d_* in Table 5 is 0.7401, and the value of *u_s_* in Table 6 is 0.7947. Therefore, based on the Equation (6), the coupling coordination degree value of the Shanghai metro system can be carried out as follows:

(20) $$D\;=\;{\left(u_{d}+u_{s}\right)}^{\frac{1}{4}}\;=\;{\left(0.7401+0.7947\right)}^{\frac{1}{4}}\;=\;0.8757$$

The calculating results for 35 cites are presented in Table 7.

**Figure 2:**
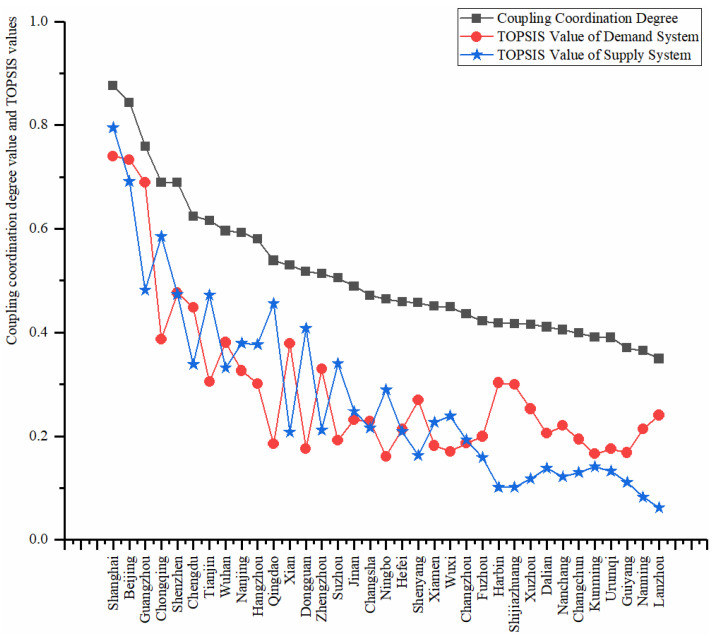
Coupling coordination degree values and TOPSIS values of all cities.

From Table 7, the average coupling coordination degree is 0.5115. And Shanghai ranks first with 0.8757, and Lanzhou lies at the end with 0.3492. According to Table 2, Beijing, Shanghai, and Guangzhou belong to the level of highly balanced, and the following 12 cities belong to the next level named barely balanced. The rest of the cities enter a slightly unbalanced level. The coupling coordination degree value and TOPSIS value of each city are shown in Figure 2.

## 5. Discussion

According to the coupling coordination degree levels and types in Table 2, the 35 cities can be classified into 6 categories in this study, as shown in Table 8.

Furthermore, combining metro scales of each metro system, 6 categories of the coupling coordination degree are further analyzed in this study. And the metro scales of the 35 cities in 2019 are shown in Table 9.

### 5.1. Highly Balanced with Lagging Demand Conditions

**Figure 3:**
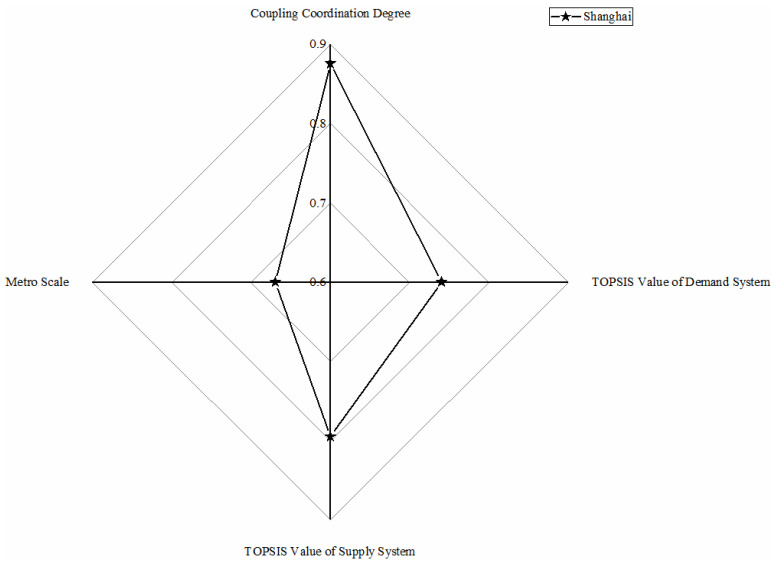
Metro in highly balanced with lagging demand conditions.

As shown in Figure 3, only the Shanghai metro system belongs to this category, whose TOPSIS values of demand and supply system and coupling coordination degree value rank first, symbolizing the most excellent development condition of the metro system. Significant population mobility, difficult environmental situation, and traffic congestion motivate the demand for metro development; meanwhile, a thriving economy, employment-population, and mature urban construction are supplied for metro development. High-levelled and coordinated conditions of demand and supply system make Shanghai a mature metro scale. The Shanghai Metro is officially the most extended railway network in the world [56].

### 5.2. Highly Balanced with Lagging Supply Conditions

**Figure 4:**
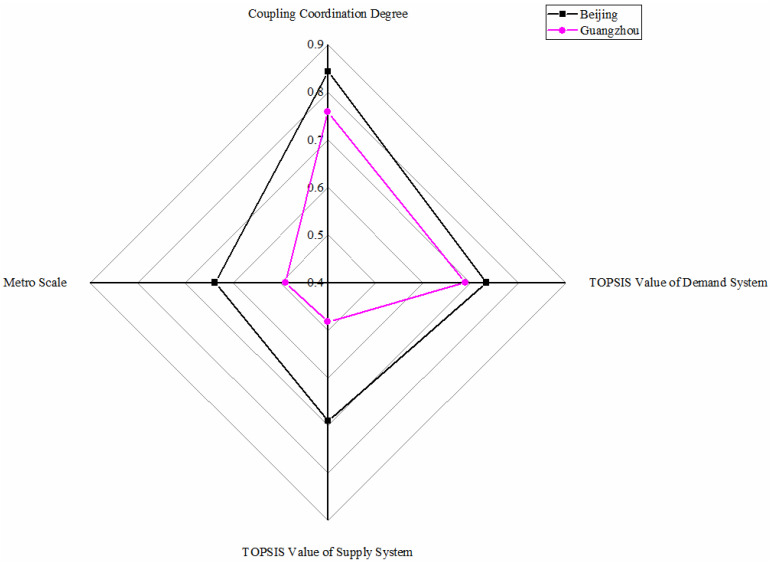
Metros in highly balanced with lagging supply conditions.

As shown in Figure 4, Beijing metro and Guangzhou metro are highly balanced with lagging supply conditions. Beijing metro shows a great development environment close to Shanghai due to its high TOPSIS demand, supply system values, and coupling coordination degree value. However, in the TOPSIS value of supply system, there is a relatively massive distance between Beijing metro and Shanghai metro mainly because of general public budget revenue, new urban workers, and construction land, which causes Beijing metro and Shanghai metro not to be in the same category. Similarly, the Beijing metro is the world’s busiest in annual ridership [57], so Beijing metro also should aim to achieve metro sustainable development. As for Guangzhou metro, it is evident that it has just reached the level of highly balanced. Guangzhou metro ranks in the forefront in all four aspects, showing a higher level from the ranking point. However, Guangzhou metro performs not so from the point of value, particularly in the TOPSIS value of the supply system. Compared with Beijing and Shanghai, also in the level of highly balanced, Guangzhou metro has disadvantages in various factors. High demand and a relatively lower supply indicates Guangzhou metro is still in the rising period of metro development [58], so Guangzhou should focus on the economy, employment, and urban construction at the city level while developing the metro to lie at the highly balanced level steadily.

### 5.3. Barely Balanced with Lagging Demand Conditions

**Figure 5:**
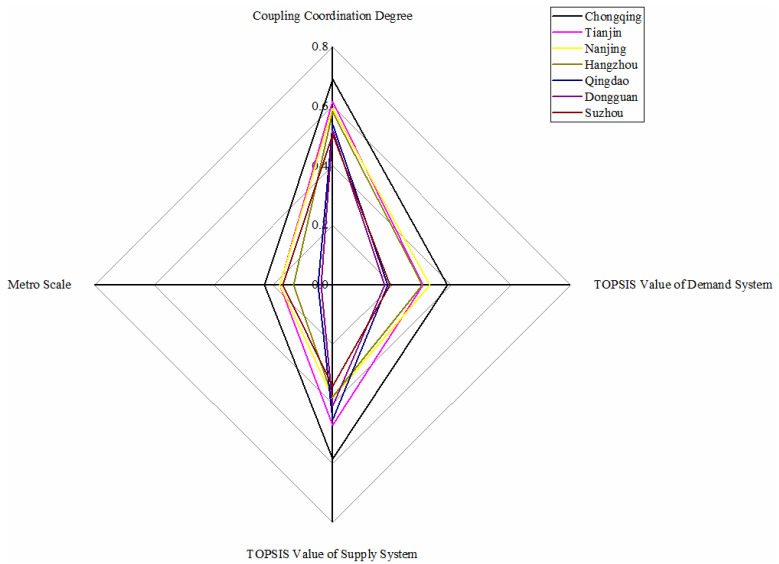
Metros in barely balanced with lagging demand conditions.

There are seven metro systems in this category: Chongqing, Tianjin, Nanjing, Hangzhou, Qingdao, Dongguan, and Suzhou, as shown in Figure 5. Chongqing metro stands first from the dimension of demand, followed by Nanjing, Tianjin, and Hangzhou; then, Suzhou, Qingdao and Dongguan are in last with a significant distance. Moreover, from the supply dimension, Chongqing metro leads the six other metro systems with apparent advantages. Chongqing, Tianjin, Nanjing, and Hangzhou rank the top four from the dimension of coupling coordination degree, and Qingdao, Dongguan, and Suzhou are relatively close behind.

Furthermore, for Chongqing metro system, its excellent supply conditions can suit the high demand well. Since 2005, Chongqing has made tremendous progress in building, facilities, and people satisfaction [59]. Under the same supply conditions, the demand of Tianjin metro is relatively low due to the insufficient support of passenger flow. Lan [60] researched that metro commuters are not the most popular form of transportation for Tianjin residents due to the higher cost of metro tickets, general walkable neighborhoods, and old metro stations’ inconvenience. However, the metro scales of Chongqing and Tianjin are small, which is related to the fact that there are other lines named monorail or light rail. Nanjing metro’s demand and supply system evaluations are pretty close, so the coupling degree is the highest. However, limited by population, economy, and construction, its coupling coordination degree does not perform as well as the coupling degree but is still at the forefront. It is pointed out by Yuan et al. [61] that Nanjing has been ranked sixth in evaluating “Cities with the Strongest Comprehensive Strength” issued by the National Statistics Bureau. Hangzhou metro and Suzhou metro are steadily advancing by taking Nanjing metro as a template through its TOPSIS value, coupling coordination value, and metro scale. Besides, Qingdao metro and Dongguan metro perform worse in the demand system with excellent supply conditions due to the underdeveloped metro scale. In the future, Chongqing and Tianjin can vigorously promote the construction of the metro based on a particular economy. Given the relatively good metro scale in Nanjing, Hangzhou, and Suzhou, steady progress will align with the current situation. Qingdao and Dongguan should pay attention to the economic situation as appropriation construction of the metro system.

### 5.4. Barely Balanced with Lagging Supply Conditions

**Figure 6:**
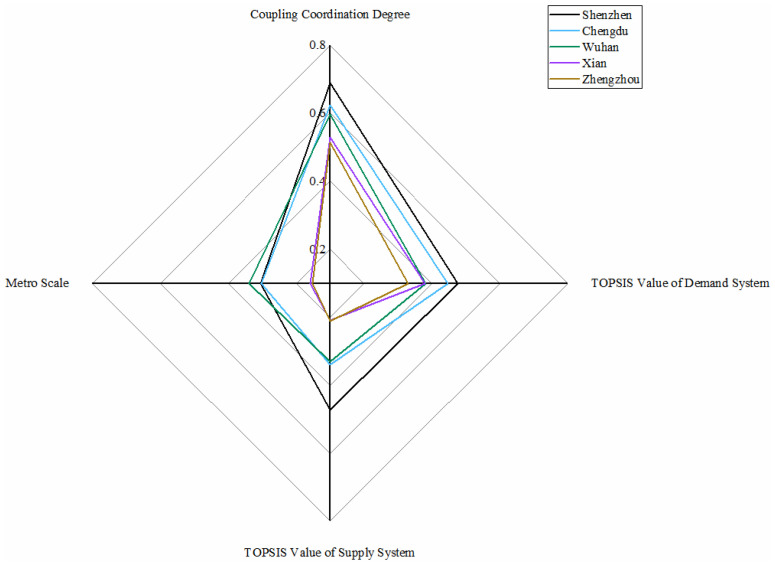
Metros in barely balanced with lagging supply conditions.

From Figure 6, Shenzhen metro system performs best in the TOPSIS value of demand and supply system and coupling coordination degree value in this category. However, Shenzhen does not perform so well for the metro scale. In Guangdong Province, Guangzhou and Shenzhen are considered the forefront of city centrality in transportation and economic networks [62]. Nevertheless, in coupling coordination conditions of metro development, there seem to be certain distances between Shenzhen and Guangzhou, mainly due to the lower demand system in Shenzhen metro system. The reason may be that there is a mutually inhibiting effect between the demand system and the metro scale.

The levels of Chengdu metro and Wuhan metro are lower than Shenzhen in most aspects. As for Chengdu, slightly weak environmental issues and adequate passenger supports make the evaluation value of the demand system a little bit higher than the supply system. Chengdu performs terribly in urban road emissions as one of the three Chinese cities with the most vehicles nationwide [63]. But for Wuhan, undeveloped urban construction makes the supply system a low level. Therefore, there is still a certain distance compared with the ultimate excellence level, the same conclusion in public transport [48]. In addition, Xian and Zhengzhou perform a high demand situation because of the worse environmental issues and crowded metro situation. In the future, the five cities should focus on developing the economy and urban construction when developing the metro to assure coordinated development of demand and supply systems.

### 5.5. Slightly Unbalanced with Lagging Demand Conditions

**Figure 7:**
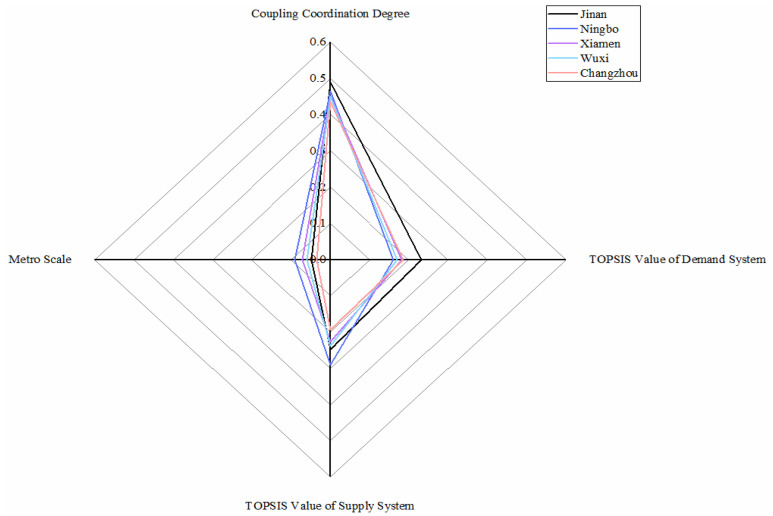
Metros in slightly unbalanced with lagging demand conditions.

As shown in Figure 7, there are five metro systems in this category. All systems are at a low level in the dimension of demand. For the dimension of supply, Ningbo metro performs best due to its economy, and four other cities are not much different. As for the value of coupling coordination degree, Jinan metro takes first, Ningbo, Xiamen and Wuxi, and Changzhou takes last.

The population is the main reason why the low evaluation values of the demand system in the cities. The resident population and passenger flow of public transportation cannot offer the development of metro supports. Environmental issues and infrastructure situations perform relatively better. In the future, the cities should focus on developing demand conditions, for example, attracting more population by developing the economy and urban construction or improving metro to attract passengers. Meanwhile, not bringing burden to the local economy should taken into consideration when developing metro.

### 5.6. Slightly Unbalanced with Lagging Supply Conditions

**Figure 8:**
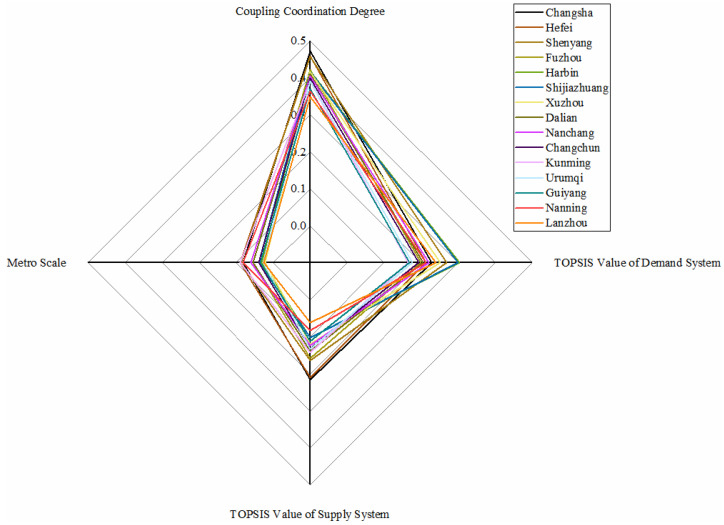
Metros in slightly unbalanced with lagging supply conditions.

As shown in Figure 8, more than one-third of the metro systems belong to this category. All the metro systems in this category are at a low level in the dimension of supply. However, in the dimension of demand, the differences between metro systems are significant. Meanwhile, the values of coupling coordination degree are between 0.35 and 0.47, which is a small distance. The economy is the main factor that pulls the evaluation values of the supply system down. Its general public budget revenue and GDP both perform worse in all the cities. That is, the economy of the cities cannot create a favourable economic environment for the development of a metro.

Moreover, some metro systems performed a high demand situation, especially Harbin metro and Shijiazhuang metro, because of the large population and the worse environmental issues. In the future, metro systems in this category should pay more attention to the development of the economy. Only in a favorable economic environment can the metro make a significant development.

## 6. Conclusions

The coupling coordination degree of 35 cities in China is measured by the indicator system and coupling coordination degree model established in this study. From the results and the discussion, firstly, the comprehensive evaluation indicator system of metro development conditions and the coupling coordination degree model can be well used to evaluate the coupling and coordination of the metro system. Secondly, the development conditions of the metro system among the 35 cities in China are quite different according to the calculation results due to the individual differences in the specific socio-economic condition in all the cities. Finally, the category of coupling coordination degree and the comparison of demand and supply conditions can offer specific suggestions for metro development in the future. For example, the metro systems of slightly unbalanced cities should put urban development first because only favorable demand and supply conditions will benefit the metro development. Moreover, cities’ metro systems in the types of barely balanced and highly balanced should improve the demand or supply conditions based on the results, keep harmony between the two systems, and finally realize sustainable development for the metro system.

This study has yet to consider the availability of data for other countries in the constructed model, which is a limitation. However, suppose the constructed model is applied to other cases. In that case, the parameters of the model will be appropriately modified according to the data characteristics of other countries. This is also the following research focus of the research team, through optimizing the established model and collecting more data for analysis of more metro systems in the world. In addition, sustainable development for the metro system is not only the improvement in demand and supply conditions, but also the sustainability parameters regarding risk management, the environmental impacts of its construction, the comparison with other mass transport systems with lower emissions or fewer infrastructure requirements, and the damage from natural disasters. Hence, there is still considerable space for improvement for model parameters to analyze the sustainable development of the metro system.

## Figures and Tables

**Table 1:** Comprehensive evaluation indicator system of metro development conditions.

Standard Layer	Factor Layer	Indicator Layer	Indicator Direction	Unit	Supporting Literature References
Demand (D)	Satisfaction of people’s going out (D1)	Resident population (D11)	+	10,000 persons	[32]
		Population density (D12)	+	persons per km^2^	[33]
		Total number of public travel passengers (D13)	+	100 million persons	[34]
	Improvement of urban environmental conditions (D2)	Average annual concentration of SO_2_ (D21)	+	µg/m^3^	[33,35]
		Average annual concentration of fine particles (PM2.5) (D22)	+	µg/m^3^	[33,35]
		The number of days that the air quality is over the second level (D23)	−	days	[33,35]
		Noise level (D24)	+	dB	[33]
	Improvement of urban traffic conditions (D3)	Congestion Index (D31)	+	-	[33]
		Per capita urban road area (D32)	−	m^2^	[32]
		Buses per 10,000 persons (D33)	−	vehicles per 10,000 persons	[32,33]
		Taxis per 10,000 persons (D34)	−	vehicles per 10,000 persons	[32,33]
		Private cars per 10,000 persons (D35)	−	10,000 vehicles per 10,000 persons	[33]
	Perfection of the development of metro (D4)	Passenger transport intensity of the network (D41)	+	10,000 persons per km-day	[36]
		The 10,000-person length of the operation network (D42)	−	kilometer per 10,000 persons	[36]
		Peak hour maximum passenger travel volume (D43)	+	10,000 persons per hour	[33,36]
		Average daily passenger travel volume (D44)	+	10,000 persons	[36]
Supply (S)	Finance (S1)	General public budget revenue (S11)	+	100 million CNY	[37]
		Gross Domestic Product (S12)	+	100 million CNY	[38]
		Per capital disposable income of urban residents (S13)	+	CNY	[38]
		Fixed asset investment (S14)	+	100 million CNY	[38]
	Manpower (S2)	Population urbanization rate (S21)	+	%	[35]
		New urban workers (S22)	+	10,000 persons	[37,39]
		Unemployment rate (S23)	−	%	[37,39]
	Land (S3)	Built-up area (S31)	+	km^2^	[38]
		Construction land (S32)	+	km^2^	[38]

“+” represents the positive indicators, while “−” represents the negative indicators.

**Table 2:** Coupling coordination degree levels and types.

Levels		Types	
Value of *D*	Development Stages	Comparison of *u*	Development Stages
0.75 ≤ *D* ≤ 1	Highly balanced	*u_d_* < *u_s_*	Highly balanced with lagging *u_d_*
		*u_d_* = *u_s_*	Highly balanced
		*u_d_* > *u_s_*	Highly balanced with lagging *u_s_*
0.5 ≤ *D* < 0.75	Barely balanced	*u_d_* < *u_s_*	Barely balanced with lagging *u_d_*
		*u_d_* = *u_s_*	Barely balanced
		*u_d_* > *u_s_*	Barely balanced with lagging *u_s_*
0.25 ≤ *D* < 0.5	Slightly unbalanced	*u_d_* < *u_s_*	Slightly unbalanced with lagging *u_d_*
		*u_d_* = *u_s_*	Slightly unbalanced
		*u_d_* > *u_s_*	Slightly unbalanced with lagging *u_s_*
0 ≤ *D* < 0.25	Seriously unbalanced	*u_d_* < *u_s_*	Seriously unbalanced with lagging *u_d_*
		*u_d_* = *u_s_*	Seriously unbalanced
		*u_d_* > *u_s_*	Seriously unbalanced with lagging *u_s_*

“*D*” represents the coupling coordination degree value, while “*u*” represents the comprehensive values of demand system and supply system.

**Table 3:** Entropy weights of demand system.

Standard Layer	Factor Layer		Indicator Layer	
	Code	Weight	Code	Weight
Demand	D1	0.2812	D11	0.0681
			D12	0.0690
			D13	0.1440
	D2	0.1942	D21	0.0537
			D22	0.0605
			D23	0.0551
			D24	0.0250
	D3	0.1567	D31	0.0255
			D32	0.0312
			D33	0.0350
			D34	0.0192
			D35	0.0458
	D4	0.3679	D41	0.0509
			D42	0.0205
			D43	0.0831
			D44	0.2134

**Table 4:** Entropy weights of supply system.

Standard Layer	Factor Layer		Indicator Layer	
	Code	Weight	Code	Weight
Supply	S1	0.5264	S11	0.2009
			S12	0.1289
			S13	0.1111
			S14	0.0855
	S2	0.2382	S21	0.0258
			S22	0.1688
			S23	0.0436
	S3	0.2354	S31	0.1583
			S32	0.0771

**Table 5:** TOPSIS values of demand system.

City	Demand System			Rank
	$\mathrm{Positive}\;\mathrm{Ideal}\;\mathrm{Distance}\;({d^{+}}_{i})$	$\mathrm{Negative}\;\mathrm{Ideal}\;\mathrm{Distance}\;({d^{-}}_{i})$	$\mathrm{Relative}\;\mathrm{Closeness}\;\mathrm{Value}\;(u_{d})$	
Shanghai	0.0965	0.2749	0.7401	1
Beijing	0.1049	0.2881	0.7332	2
Guangzhou	0.1078	0.2392	0.6893	3
Shenzhen	0.1788	0.1624	0.4759	4
Chengdu	0.1835	0.1490	0.4481	5
Chongqing	0.2080	0.1314	0.3870	6
Wuhan	0.2009	0.1235	0.3808	7
Xian	0.2133	0.1298	0.3782	8
Zhengzhou	0.2452	0.1204	0.3293	9
Nanjing	0.2216	0.1073	0.3262	10
Tianjin	0.2418	0.1060	0.3048	11
Harbin	0.2646	0.1149	0.3027	12
Hangzhou	0.2356	0.1016	0.3014	13
Shijiazhuang	0.2718	0.1161	0.2993	14
Shenyang	0.2539	0.0936	0.2694	15
Xuzhou	0.2860	0.0965	0.2523	16
Lanzhou	0.2787	0.0882	0.2405	17
Jinan	0.2858	0.0861	0.2314	18
Changsha	0.2638	0.0781	0.2286	19
Nanchang	0.2782	0.0785	0.2201	20
Nanning	0.2732	0.0743	0.2138	21
Hefei	0.2718	0.0736	0.2132	22
Dalian	0.2716	0.0701	0.2051	23
Fuzhou	0.2897	0.0722	0.1994	24
Changchun	0.2784	0.0671	0.1942	25
Suzhou	0.2685	0.0637	0.1918	26
Changzhou	0.2933	0.0671	0.1862	27
Qingdao	0.2747	0.0623	0.1849	28
Xiamen	0.2909	0.0646	0.1817	29
Dongguan	0.2915	0.0622	0.1758	30
Urumqi	0.2918	0.0621	0.1755	31
Wuxi	0.2895	0.0594	0.1703	32
Guiyang	0.2949	0.0595	0.1680	33
Kunming	0.2833	0.0563	0.1657	34
Ningbo	0.2855	0.0546	0.1607	35

**Table 6:** TOPSIS values of supply system.

City	Supply System			Rank
	$\mathrm{Positive}\;\mathrm{Ideal}\;\mathrm{Distance}\;({d^{+}}_{i})$	$\mathrm{Negative}\;\mathrm{Ideal}\;\mathrm{Distance}\;({d^{-}}_{i})$	$\mathrm{Relative}\;\mathrm{Closeness}\;\mathrm{Value}\;(u_{s})$	
Shanghai	0.0849	0.3288	0.7947	1
Beijing	0.1305	0.2919	0.6910	2
Chongqing	0.1916	0.2701	0.5850	3
Guangzhou	0.2143	0.1994	0.4821	4
Shenzhen	0.2109	0.1897	0.4736	5
Tianjin	0.2085	0.1864	0.4720	6
Qingdao	0.2386	0.1996	0.4554	7
Dongguan	0.2541	0.1756	0.4086	8
Nanjing	0.2440	0.1488	0.3789	9
Hangzhou	0.2439	0.1473	0.3766	10
Suzhou	0.2692	0.1388	0.3401	11
Chengdu	0.2557	0.1312	0.3390	12
Wuhan	0.2562	0.1273	0.3318	13
Ningbo	0.2880	0.1174	0.2896	14
Jinan	0.2926	0.0964	0.2478	15
Wuxi	0.3111	0.0975	0.2386	16
Xiamen	0.3102	0.0912	0.2272	17
Changsha	0.3098	0.0853	0.2160	18
Zhengzhou	0.3056	0.0819	0.2113	19
Hefei	0.3029	0.0801	0.2091	20
Xian	0.3068	0.0806	0.2080	21
Changzhou	0.3339	0.0801	0.1934	22
Shenyang	0.3237	0.0629	0.1627	23
Fuzhou	0.3298	0.0623	0.1588	24
Kunming	0.3258	0.0537	0.1414	25
Dalian	0.3318	0.0533	0.1385	26
Urumqi	0.3363	0.0512	0.1322	27
Changchun	0.3383	0.0507	0.1303	28
Nanchang	0.3443	0.0480	0.1224	29
Xuzhou	0.3496	0.0469	0.1183	30
Guiyang	0.3409	0.0428	0.1116	31
Shijiazhuang	0.3449	0.0388	0.1012	32
Harbin	0.3434	0.0385	0.1008	33
Nanning	0.3561	0.0322	0.0829	34
Lanzhou	0.3625	0.0239	0.0619	35

**Table 7:** Coupling Coordination Degree Values.

City	Metro Development Conditions Comprehensive Evaluation Indicator System	Rank
	D	
Shanghai	0.8757	1
Beijing	0.8437	2
Guangzhou	0.7592	3
Chongqing	0.6898	4
Shenzhen	0.6890	5
Chengdu	0.6243	6
Tianjin	0.6159	7
Wuhan	0.5962	8
Nanjing	0.5929	9
Hangzhou	0.5805	10
Qingdao	0.5387	11
Xian	0.5296	12
Dongguan	0.5177	13
Zhengzhou	0.5136	14
Suzhou	0.5054	15
Jinan	0.4894	16
Changsha	0.4714	17
Ningbo	0.4645	18
Hefei	0.4595	19
Shenyang	0.4576	20
Xiamen	0.4508	21
Wuxi	0.4490	22
Changzhou	0.4356	23
Fuzhou	0.4219	24
Harbin	0.4179	25
Shijiazhuang	0.4172	26
Xuzhou	0.4157	27
Dalian	0.4105	28
Nanchang	0.4051	29
Changchun	0.3988	30
Kunming	0.3913	31
Urumqi	0.3903	32
Guiyang	0.3700	33
Nanning	0.3648	34
Lanzhou	0.3492	35

**Table 8:** Categories of metro systems.

Types of Metro Systems		Cities
Highly Balanced	Lagging Demand Conditions	Shanghai
	Lagging Supply Conditions	Beijing, Guangzhou
Barely Balanced	Lagging Demand Conditions	Chongqing, Tianjin, Nanjing, Hangzhou, Qingdao, Dongguan, Suzhou
	Lagging Supply Conditions	Shenzhen, Chengdu, Wuhan, Xian, Zhengzhou
Slightly Unbalanced	Lagging Demand Conditions	Jinan, Ningbo, Xiamen, Wuxi, Changzhou
	Lagging Supply Conditions	Changsha, Hefei, Shenyang, Fuzhou, Harbin, Shijiazhuang, Xuzhou, Dalian, Nanchang, Changchun, Kunming, Urumqi, Guiyang, Nanning, Lanzhou

**Table 9:** Metro scales.

City	Metro Scales (kkm)	City	Metro Scales (kkm)	City	Metro Scales (kkm)
Beijing	0.6376	Xian	0.1580	Fuzhou	0.0534
Shanghai	0.6695	Harbin	0.0303	Dongguan	0.0378
Tianjin	0.1786	Suzhou	0.1659	Nanning	0.0809
Chongqing	0.2300	Zhengzhou	0.1517	Hefei	0.0895
Guangzhou	0.4894	Kunming	0.0887	Shijiazhuang	0.0384
Shenzhen	0.3044	Hangzhou	0.1309	Guiyang	0.0348
Wuhan	0.3384	Changsha	0.0818	Xiamen	0.0719
Nanjing	0.1768	Ningbo	0.0913	Urumqi	0.0268
Shenyang	0.0872	Wuxi	0.0588	Jinan	0.0477
Changchun	0.0387	Nanchang	0.0604	Changzhou	0.0342
Dalian	0.0541	Lanzhou	0.0255	Xuzhou	0.0218
Chengdu	0.3022	Qingdao	0.0500		

“kkm” represents the thousand kilometres.

## Data Availability

The data presented in this study are available in the main text of the article.

## Appendix A. Original Data

**Table A1:** Data of Demand System.

City	Indicator Layer															
	D11	D12	D13	D21	D22	D23	D24	D31	D32	D33	D34	D35	D41	D42	D43	D44
Beijing	2154	1137	70.73	4	42	240	69.6	2.040	7.68	10.68	33.21	0.2310	1.56	0.358	6.32	1086.9
Shanghai	2428	3830	59.17	7	35	309	68.3	1.739	4.72	7.37	16.46	0.1400	1.51	0.334	5.95	1064.3
Tianjin	1562	4939	16.16	11	51	219	66.5	1.612	12.98	8.16	20.45	0.1683	0.60	0.153	1.76	144.2
Chongqing	2027	2012	34.93	7	38	316	64.6	2.165	14.38	7.04	11.72	0.1989	0.87	0.162	3.77	285.4
Guangzhou	1531	6141	55.36	7	30	293	69.3	1.744	13.90	9.97	13.21	0.1417	1.74	0.327	4.86	906.8
Shenzhen	1344	6728	35.50	5	24	332	69.7	1.600	8.46	12.73	16.00	0.2031	1.66	0.235	5.38	490.8
Wuhan	1121	6438	26.49	9	45	245	68.7	1.716	13.22	8.67	15.87	0.2735	0.88	0.346	3.34	340.5
Nanjing	850	1588	20.66	10	40	255	67.4	1.705	24.30	10.32	14.22	0.2485	0.84	0.464	4.01	315.7
Shenyang	832	3535	14.63	21	43	284	70.0	1.677	15.39	7.17	26.24	0.2581	0.60	0.222	2.46	110.6
Changchun	773	1349	9.09	11	38	306	69.5	1.777	16.52	6.58	20.94	0.2127	0.56	0.152	1.16	56.1
Dalian	699	2899	11.39	11	35	302	67.3	1.659	13.86	8.04	16.65	0.2123	0.35	0.259	2.66	55.7
Chengdu	1658	6357	28.76	6	43	287	69.2	1.610	15.87	9.62	9.97	0.2647	1.22	0.263	5.57	417.6
Xian	1020	6767	23.20	9	58	225	70.5	1.730	18.44	10.36	16.50	0.3021	1.65	0.155	3.58	260.4
Harbin	1076	10,542	11.65	17	42	304	71.8	1.905	15.87	6.88	16.71	0.1660	0.94	0.028	1.29	28.4
Suzhou	1075	2520	11.57	9	36	288	66.4	1.483	29.66	8.34	8.39	0.3260	0.60	0.195	2.11	100.2
Zhengzhou	1035	8793	13.45	9	58	177	67.8	1.580	9.39	6.10	10.54	0.3380	0.84	0.188	2.99	126.7
Kunming	695	2282	9.82	12	26	356	67.4	1.676	9.83	9.82	13.29	0.3235	0.66	0.128	1.52	58.6
Hangzhou	1036	3950	20.41	7	38	287	68.6	1.627	13.69	10.34	13.64	0.2087	1.36	0.126	3.71	177.9
Changsha	839	3207	10.18	7	47	275	69.4	1.687	13.91	11.43	9.29	0.2841	1.01	0.120	2.25	101.2
Ningbo	854	2371	5.91	9	29	318	68.9	1.380	12.86	8.28	7.34	0.2770	0.53	0.113	1.28	51.1
Wuxi	659	2089	4.64	8	39	263	68.6	1.443	27.56	7.15	7.82	0.2613	0.51	0.089	0.83	30.0
Nanchang	560	6804	5.53	9	35	322	66.7	1.499	13.49	6.99	9.74	0.1786	0.80	0.108	1.47	48.0
Lanzhou	379	7817	8.75	18	36	296	68.8	1.548	21.54	8.20	27.87	0.1840	0.66	0.228	1.21	16.9
Qingdao	950	1714	12.69	8	37	287	68.3	1.574	19.32	8.94	11.45	0.2663	0.28	0.194	2.13	51.7
Fuzhou	780	4131	5.41	5	24	360	68.7	1.613	13.65	4.91	8.13	0.1526	0.63	0.068	1.04	33.5
Dongguan	846	3886	3.40	10	32	285	69.3	1.588	12.88	7.07	4.01	0.3385	0.39	0.045	0.95	14.7
Nanning	734	4386	6.00	10	33	333	69.0	1.526	20.94	5.12	9.29	0.2104	0.99	0.110	1.87	80.0
Hefei	819	3893	7.41	6	44	254	68.1	1.712	19.5	9.76	14.11	0.2282	0.77	0.109	1.79	68.5
Shijiazhuang	1039	6491	4.68	16	63	174	66.9	1.593	18.54	3.74	6.46	0.2491	0.68	0.037	1.44	26.2
Guiyang	497	2412	5.98	10	27	358	69.8	1.979	10.25	6.41	21.01	0.2408	0.40	0.070	0.88	13.9
Xiamen	429	8437	8.32	6	24	356	67.2	1.666	28.5	10.31	13.24	0.2709	0.42	0.168	0.95	30.1
Urumqi	355	4153	7.41	9	50	277	64.9	1.517	18.92	12.42	36.88	0.2830	0.32	0.075	0.56	8.6
Jinan	891	2546	7.63	15	53	207	69.6	1.684	20.53	9.53	12.68	0.2618	0.10	0.054	0.33	5.0
Changzhou	474	2946	3.05	10	44	255	67.5	1.248	21.87	5.60	7.77	0.2546	0.28	0.072	0.70	9.6
Xuzhou	883	3432	3.38	11	57	216	69.1	1.519	23.04	4.92	7.75	0.1581	0.36	0.025	0.45	7.8

**Table A2:** Data of Supply System.

City	Indicator Layer								
	S11	S12	S13	S14	S21	S22	S23	S31	S32
Beijing	5817.10	35,371.30	73,849	7868.74	86.6	35.10	4.1	1469	1472
Shanghai	7165.10	38,155.32	73,615	8021.20	88.1	58.91	3.6	1238	1945
Tianjin	2410.25	14,104.28	46,119	12,122.73	83.5	50.17	3.5	1151	998
Chongqing	2134.90	23,605.77	37,939	19,725.11	66.8	75.16	2.6	1515	1319
Guangzhou	1697.21	23,628.60	65,052	7462.12	86.5	33.73	2.2	1324	722
Shenzhen	3773.21	26,927.09	62,522	7374.71	100.0	16.03	2.2	960	940
Wuhan	1564.12	16,223.21	51,706	9559.25	80.5	24.25	2.0	812	865
Nanjing	1580.03	14,030.15	64,372	7343.38	83.2	32.52	1.8	823	802
Shenyang	730.30	6470.30	46,786	1936.91	81.0	11.89	2.9	563	630
Changchun	420.00	5904.10	37,844	4489.71	49.6	10.20	2.5	543	537
Dalian	692.80	7001.70	46,468	1459.43	78.7	11.60	2.7	444	439
Chengdu	1483.00	17,012.65	45,878	9175.21	74.4	26.40	3.3	950	879
Xian	702.56	9321.19	41,850	8281.32	74.6	16.12	3.3	701	701
Harbin	370.90	5249.40	40,007	5372.54	65.9	10.10	3.5	446	438
Suzhou	2221.80	19,235.80	68,629	4933.10	77.0	17.32	1.8	478	476
Zhengzhou	1222.50	11,589.70	42,087	8634.07	74.6	11.50	1.8	581	563
Kunming	630.03	6475.88	46,289	4574.52	73.6	16.15	3.4	446	458
Hangzhou	1966.00	15,373.05	66,068	7242.34	78.5	34.00	1.8	648	612
Changsha	950.23	11,574.22	55,211	9290.31	79.6	14.77	2.7	378	324
Ningbo	1468.50	11,985.00	64,886	5610.33	73.6	25.40	1.6	355	397
Wuxi	1036.33	11,852.32	61,915	3595.94	77.1	15.42	1.8	347	300
Nanchang	476.08	5596.18	44,136	6253.42	75.2	7.54	2.3	356	319
Lanzhou	233.23	2837.36	38,095	1405.33	81.0	9.36	3.4	314	322
Qingdao	1247.10	11,741.31	54,484	10,204.05	74.1	75.10	3.0	758	651
Fuzhou	668.08	9392.30	47,920	7090.15	70.5	13.55	2.2	301	225
Dongguan	673.18	9482.50	55,156	2128.43	92.1	44.71	1.2	1194	1194
Nanning	370.93	4506.56	37,675	5293.10	63.7	7.52	2.7	320	315
Hefei	745.99	9409.40	45,404	7414.60	76.3	27.76	2.8	481	464
Shijiazhuang	569.10	5809.90	38,550	7192.38	65.1	13.90	3.2	309	57
Guiyang	417.26	4039.60	38,240	4660.41	76.1	18.51	3.0	369	345
Xiamen	768.37	5995.04	59,018	2857.97	89.2	26.18	2.8	398	383
Urumqi	472.46	3413.26	42,667	2266.61	90.2	14.50	2.7	488	456
Jinan	874.20	9443.37	51,913	5385.10	71.2	19.39	2.0	716	702
Changzhou	590.00	7400.90	58,345	4423.08	73.3	11.30	1.8	273	273
Xuzhou	468.32	7151.35	36,215	5759.35	66.7	11.25	1.8	282	280
